# Risk Factors for Alzheimer’s Disease: Focus on Stress

**DOI:** 10.3389/fphar.2019.00976

**Published:** 2019-09-10

**Authors:** Alessandra Caruso, Ferdinando Nicoletti, Alessandra Gaetano, Sergio Scaccianoce

**Affiliations:** ^1^Department of Physiology and Pharmacology, Sapienza Università di Roma, Rome, Italy; ^2^Neuropharmacology Research Unit, I.R.C.C.S. Neuromed, Pozzilli, Italy

**Keywords:** stress, glucocorticoids, Alzheimer’s disease, risk factor, animal model

## Abstract

In vulnerable individuals, chronic and persistent stress is an established risk factor for disorders that are comorbid with Alzheimer’s disease (AD), such as hypertension, obesity and metabolic syndrome, and psychiatric disorders. There are no disease-modifying drugs in the treatment of AD, and all phase-3 clinical trials with anti-amyloid drugs (e.g., β- or γ-secretase inhibitors and monoclonal antibodies) did not meet the primary endpoints. There are many reasons for the lack of efficacy of anti-amyloid drugs in AD, the most likely being a late start of treatment, considering that pathophysiological mechanisms underlying synaptic dysfunction and neuronal death begin several decades before the clinical onset of AD. The identification of risk factors is, therefore, an essential step for early treatment of AD with candidate disease-modifying drugs. Preclinical studies suggest that stress, and the resulting activation of the hypothalamic–pituitary–adrenal axis, can induce biochemical abnormalities reminiscent to those found in autoptic brain samples from individuals affected by AD (e.g., increases amyloid precursor protein and tau hyperphosphorylation). In this review, we will critically analyze the current knowledge supporting stress as a potential risk factor for AD.

## Introduction

According to the World Health Organization, “a risk factor is any attribute, characteristic or exposure of an individual that increases the likelihood of developing a disease or injury” (www.who.int/topics/risk_factors). Alzheimer’s disease (AD) is a neurodegenerative disorder characterized by progressive impairments in cognitive functions (Hampel et al., 2015). AD is characterized by loss of neurons and synapses in the cerebral cortex and hippocampus (Nisticò et al., 2012). Formation of aggregates of the β-amyloid peptide (Aβ_1-42_) and neurofibrillary tangles resulting from tau protein hyperphosphorylation are the major hallmarks of AD. These histopathological processes occur in brain regions that are involved in memory formation and emotional regulation (Gómez-Isla et al., 1996; Murray et al., 2006; Holtzman et al., 2011). The hippocampus is particularly vulnerable to AD-associated neuronal damage (Mu and Gage, 2011; Hollands et al., 2016). Genetic studies of early-onset familial AD (eFAD) have demonstrated that the formation of Aβ_1-42_ aggregates, rather than tau hyperphosphorylation, lies at the core of AD. eFAD is caused by mutations in the genes encoding for amyloid-ß precursor protein (APP) (Goate, 2006), presenilin 1 (PSEN1) (Sherrington et al., 1995), and presenilin 2 (PSEN2) (Levy-Lahad et al., 1995; Rogaev et al., 1995) inherited as an autosomal dominant trait (Guerreiro et al., 2012). PSEN1 mutations account for most eFAD, while APP and PSEN2 are rarer. However, these findings have constituted the bases that led to the proposal of the so-called “amyloid cascade hypothesis,” which posits that dysregulation of amyloid-ß (Aß) peptide production and/or proteolytic degradation plays a key role in triggering the pathological and behavioral changes observed in AD patients (Selkoe and Hardy, 2016). Although our knowledge of neuropathological and neurochemical alterations associated with AD has impressively increased in the last decades, the current treatment is limited to cholinesterase inhibitors and the N-methyl-D-aspartate (NMDA) receptor channel blocker, memantine. None of these drugs can slow the progression of AD. Several putative disease-modifying drugs have been developed and continue to be developed with the hope of restraining the progression of the disease. Most of these drugs target either the production or the aggregation process of Aβ_1-42_ (Anand et al., 2017). Results of clinical studies with all these drugs have been highly disappointing. For example, a recently concluded randomized clinical trial with an inhibitor of β-secretase (BACE1), the enzyme that cleaves APP to uncover the N-terminus domain of Aβ_1-42_, did not show any reduction in cognitive or functional decline in AD patients, suggesting that either disease progression does not rely exclusively on amyloid formation or, alternatively, that anti-amyloid drugs should be administered several years prior to the onset of AD to be effective (Egan et al., 2018). The entire AD community was frustrated by the lack of efficacy of aducanumab, an anti-amyloid monoclonal antibody that was considered as highly promising based on a phase 1b clinical trial (Sevigny et al., 2016). If these drugs fail because treatment starts too late, i.e. when pathophysiological mechanisms of AD are already established, research should be directed to the identification of risk factors that can reliably predict the development of AD. As highlighted above, a minority of patients has eFAD with autosomal dominant transmission. Children have 50% chance to inherit the same mutation, and they are natural candidates for early treatment with candidate disease-modifying drugs. Apolipoprotein E4 (ApoE4) is the most established risk factor for sporadic AD (besides age), and subjects who are homozygous for ε4 (the gene encoding for ApoE4) and showed brain amyloidosis by PET scanning at an early age are also candidates for early treatment. The presence of ApoE4 may also predict responses to drug treatment in AD. For example, inhibitors of angiotensin-converting enzyme (ACE) improve cognition in patients affected by AD carrying ApoE4 and certain ACE polymorphisms (de Oliveira et al., 2014; de Oliveira et al., 2018). However, only about half of AD patients are ApoE4-positive, and the presence of cerebral amyloidosis is only suggestive of later development of AD (old individuals may have cerebral amyloidosis without AD).

Cardiovascular and metabolic disorders, such as hypertension, type-2 diabetes, metabolic syndrome, hypercholesterolemia, unhealthy dietary pattern, poor physical and cognitive activity, and smoking may increase the vulnerability to develop AD (Barnard et al., 2014; Xu et al., 2015). This review aims to comment on preclinical and clinical data on stress and glucocorticoids as risk factors for AD. Stress activates the hypothalamic–pituitary–adrenal (HPA) axis, with an ensuing increase in blood levels of glucocorticoid hormones (cortisol in humans and corticosterone in rodents). Hypothalamic corticotrophin-releasing hormone (CRH) is the main secretagogue of adrenocorticotropic hormone (ACTH) from the pituitary gland. ACTH, in turn, stimulates the production of glucocorticoids from the adrenal cortex. Glucocorticoids exert a crucial role in the adaptive physiological and behavioral responses to stress. Moreover, glucocorticoid hormones exert a negative feedback signal capable of inhibiting the activation of the HPA axis: the main targets of glucocorticoid-induced negative feedback are the anterior pituitary, the hypothalamus, and the hippocampus. Glucocorticoid binds to two receptors: the mineralocorticoid receptor (MR) and the glucocorticoid receptor (GR). Both are ligand-dependent transcription factors. Of the two receptors, MRs have one order of magnitude higher affinity for glucocorticoids than GRs. At low levels of circulating glucocorticoids, e.g., during the circadian nadir, MRs are fully occupied; in contrast, GR activation occurs at the circadian peak of glucocorticoids or in response to stressful events. Interestingly, both MRs and GRs are highly expressed in pyramidal neurons of CA1 and CA2 and in granule cells of the dentate gyrus of the hippocampus (Han et al., 2005), which is a vulnerable brain region in AD (Henneman et al., 2009). It has been hypothesized that long-lasting stress and the resulting sustained hypocortisolemia could be a potential neurodegenerative factor for the hippocampus (Angelucci, 2000). However, recent findings have depicted a more complex relationship between stress and neurodegeneration.

## Hypothalamic–Pituitary–Adrenal Axis Dysfunction In Alzheimer’s Disease

Clinical reports of hypercortisolism in AD patients suggest a causal role for glucocorticoids in AD (Bruno et al., 1995; Hatzinger et al., 1995; Greenwald et al., 1986; Peskind et al., 2001; Rasmuson et al., 2001; Wilson et al., 2003; Hoogendijk et al., 2006; Johansson et al., 2010; Curto et al., 2017; Ouanes and Popp, 2019). However, it should be considered that some degree of stress could be present in a condition involving bodily or psychic suffering, especially when patients are cognitively able to perceive memory impairment, which is among the first symptoms reported by patients suffering from AD (Saydak et al., 1987). Dysregulation of the corticotropic axis is present in individuals suffering from depression, diabetes, and metabolic syndrome. These clinical conditions have been hypothesized to increase the risk to develop AD later in life (Ownby et al., 2006; Huang et al., 2014; Rojas-Gutierrez et al., 2017). In particular, it has been reported that patients who experienced late-life, but not early- or mid-life, depression had a two-fold increased risk for AD (Barnes et al., 2012; Singh-Manoux et al., 2017). Single nucleotide polymorphism (SNP) analysis in patients affected by AD supports the hypothesis that elevated glucocorticoid levels increase the risk to develop AD. de Quervain et al. (2004) analyzed SNPs in 10 glucocorticoid-related genes in 814 AD patients. They found an association between AD and a rare haplotype in the 5’ regulatory region of the gene encoding for type-1 11ß-hydroxysteroid dehydrogenase (11ß-HSD1). 11ß-HSD1, also known as cortisone reductase, catalyzes the conversion of cortisol into the biological inert 11-keto derivative (cortisone). Thus, subjects carrying this rare haplotype with reduced 11ß-HSD1 transcription show less inactivation of glucocorticoids, which, in turn, is associated with an increased vulnerability to the clinical manifestation of AD. On the contrary, subjects bearing a polymorphism of the GR gene (NR3C1) are characterized by a reduced risk to develop AD (van Rossum et al., 2008). More precisely, carriers of the ER22/23EK allele (approximately 7% of the entire population) were associated with a decreased risk of developing dementia. The presence of the ER22/23EK allele leads to a decreased sensitivity of GRs to glucocorticoids (Russcher et al., 2005).

Several lines of evidence suggest a tight connection between neuroinflammation and AD (see Nichols et al., 2019 for a recent review). In a double-blind, placebo-controlled trial, 138 AD patients received prednisone (10 mg daily for 1 year). Glucocorticoid treatment not only failed to ameliorate cognitive decline as assessed by the Alzheimer’s Disease Assessment Scale but also caused a greater behavioral decline, as measured by the Brief Psychiatric Rating Scale (Aisen et al., 2000). These findings suggest a detrimental effect of glucocorticoids in AD. Some clinical trials have investigated the effects of the glucocorticoid receptor antagonist, mifepristone, in AD patients (Belanoff et al., 2002; DeBattista and Belanoff, 2005). Although a significant improvement of cognitive function was observed in AD patients after a 6-week treatment with 200 mg of mifepristone (Pomara et al., 2002), there are no ongoing clinical trials with glucocorticoid antagonists in AD.

## Preclinical Studies On Stress As Risk Factor For Alzheimer’s Disease

Preclinical studies aimed at elucidating the role of glucocorticoids as a risk factor for AD have been mostly conducted in transgenic (Tg) mice, such as Tg2576 mice expressing human APP carrying the Swedish mutation (KM670/671NL), mice with a double mutations of APP and PSEN1, and 3xTgAD mice, characterized by a triple mutations of APP (Swedish mutations), PSEN1 (M146V), and the P301L mutation in the gene encoding tau protein (MAPT) (Götz et al., 2018). However, it is important to note that transgenic AD mice recapitulate features of eFAD that represents only 3% of AD (Bird, 1999), with the important limitation of the limited lifespan of mice. Rats have been used for the induction of “AD-like” pathology using i.c.v. or intrahippocampal injection of Aβ_1-42_ oligomers, tau protein, or excitotoxins (see Shree et al., 2017 for a recent review). In both Tg and non-Tg models, the effect of stress was investigated by either exposing animals to stress of variable duration or administering glucocorticoids (the natural hormone, corticosterone, or the synthetic, long-acting, and GR-selective glucocorticoid, dexamethasone). Some studies have investigated the role of CRH independently of its function in the regulation of the HPA axis and the potential use of CRH receptor antagonists as disease-modifying drugs in AD (Rissman et al., 2012). One of the first demonstrations that stress hormones are linked to AD-like neuropathology was provided by the evidence that kainic acid-induced tau hyperphosphorylation was amplified by repeated (i.e., 7 days) corticosterone administration in rats (Elliott et al., 1993). Dexamethasone treatment in rats was also found to increase the expression of APP in the cerebral cortex, cerebellum, and brain stem (Budas et al., 1999). The effect of glucocorticoids on APP processing and Aβ_1-42_ production was also investigated in 3xTgAD mice, in which dexamethasone treatment for 7 days caused a significant increase in soluble and insoluble Aβ_1-42_ in the hippocampus, cortex, and amygdala, and also leads to the mislocalization of tau to the somatodendritic compartment (Green et al., 2006). Moreover, neuroblastoma N2A cells incubated with dexamethasone or corticosterone showed an increased expression of both APP and BACE leading to enhanced production of Aβ_1-42_. Interestingly, 3xTgAD mice showed an age-dependent increase in serum corticosterone levels, which is observable already at 9 months of age (Green et al., 2006). Although glucocorticoid administration mimics only partially the hormonal endpoint of stress-induced HPA activation, the above findings paved the way to explore the effect of stress (of different intensity and duration) on AD neuropathology. One of the most popular Tg mouse models of AD expresses human APP with the London mutation (V717I). Using this model, it has been demonstrated that exposure to long-term (8 months) restraint stress caused learning and memory deficits as well as an increase in extracellular amyloid plaque deposition and intraneuronal APP and Aβ_1-42_ immunoreactivity, and neurodegeneration in the hippocampus and cerebral cortex (Jeong et al., 2006). Tg2576 transgenic mice expressing human APP with the Swedish mutation (K670M/N671L) were used to study the effects restraint stress (2 h daily for 16 consecutive days) (Lee et al., 2009). Stress caused a rapid increase in plaque formation, insoluble Aβ accumulation, and dendritic atrophy of cortical neurons (Lee et al., 2009). Besides, restraint stress caused a down-regulation of matrix metalloproteinase-2 (MMP-2), which, similarly to MMP-9, is involved in the clearance of Aβ (Roher et al., 1994). In the same study, the authors have demonstrated that MMP-2 down-regulation and Aβ pathology were completely prevented by the administration of the CRH receptor antagonist, NBI 27914, reinforcing the hypothesis that over-activation of the HPA axis contributes to the development of stress-induced AD-like pathology. The hypothesis that a down-regulation of MMP-2 is a linking bridge between stress and AD pathology is supported by the evidence that i) MMP-2 expression was reduced in cortical neurons treated with corticosterone (Lee et al., 2009); ii) infusion of the MMP inhibitor, GM6001, increases Aβ formation in Tg2576 mice (Yin et al., 2006); and iii) MMP-2 was reduced in the parietal cortex of Tg2576 mice (Lee et al., 2009).

Tau hyperphosphorylation is a molecular hallmark present in both the hereditary and sporadic forms of AD. Hyperphosphorylated tau protein has a key pathogenic role in AD neuronal dysfunction because it accumulates in form of insoluble aggregates and neurofibrillary tangles with a consequent malfunction of axonal transport (Iqbal et al., 2010; Fitzpatrick et al., 2017). When exposed to dexamethasone, neuronal cell lines bioengineered to express the human homolog of the protein tau (PC12-htau) showed a greater degree of susceptibility to the neurotoxic actions of Aβ_1-42_ as well as marked increases in tau hyperphosphorylation at specific epitopes implicated in AD neuropathology. More specifically, exposure to dexamethasone reduced tau turnover and, consequently, increased cytoplasmic accumulation of tau. These effects were abolished by pharmacological blockade of GRs with mifepristone, indicating that activation of GRs mediates the effects of glucocorticoids on tau protein. Tau hyperphosphorylation was ultimately mediated by a GR-dependent activation of cyclin-dependent kinase 5 (CDK5) and glycogen synthase kinase-3β (GSK3β) (Sotiropoulos et al., 2008). The effect of stress on tau hyperphosphorylation has been extensively studied in recent years. Sotiropoulos et al. (2011) found that in Wistar rats, exposure to unpredictable chronic (1 month) stress induced tau hyperphosphorylation in the hippocampus and prefrontal cortex. In line with the hypothesis that glucocorticoids mediate the action of stress, these authors also demonstrated that treatment with dexamethasone for 14 days mimicked the amplifying effect of stress on Aβ-induced tau hyperphosphorylation. Both stress and glucocorticoid administration activated GSK3β and CDK5, as well as calcium-calmodulin-dependent protein kinase-II, the MAP kinase pathway, and the JUN kinase pathway in the hippocampus and prefrontal cortex, and caused an impairment in the hippocampus- and prefrontal cortex-dependent memory. Based on these findings, the authors concluded that sustained stress, *via* glucocorticoid hypersecretion, could influence the onset and progression of AD pathology and highlighted the role of tau hyperphosphorylation in the effect of stress on AD. CRF is the principal driving force, which controls both tonic and phasic activation of the HPA axis. However, the hypothesis that CRF might have a causative role in AD independently of ACTH and glucocorticoid secretion has been addressed in several studies. Rissman and colleagues have demonstrated that stress-induced tau hyperphosphorylation was not prevented by adrenalectomy, while it was absent in type-1 CRF receptor (CRFR1)-deficient mice and mice treated with a selective CRFR1 antagonist (antalarmin). This suggested that CRF induced tau pathology through a central mechanism independent of the activation of the HPA axis (Rissman et al., 2007). They used two mouse models of AD, i.e., Tg2576 mice, which express APPK670/671L, and PS19 mice, which express human P301S mutant tau. They also used two different stress protocols: chronic restraint stress (CRS) and chronic unpredictable stress (CUS), both delivered for 1 month. In both Tg2576 and PS19 mice, CRS, but not CUS, induced an increase in Aβ_1-42_ and hyperphosphorylated tau in the hippocampus and frontal cortex. Moreover, CRS, but not CUS, caused deficits in hippocampus-dependent memory. In apparent contrast with the glucocorticoid-centric hypothesis of stress and AD, PS19 mice implanted with a corticosterone pellet did not show increases in the levels of hyperphosphorylated tau. In contrast, injection of the CRF antagonist, NBI 27914, 15 min before the onset of restraint stress abolished tau accumulation and prevented memory impairment. The hypothesis of a central action of CRF in causing AD-like neuropathology was further supported by the demonstration that transgenic mice overexpressing CRF showed an increase in tau phosphorylation in the hippocampus, and CRFR1 ablation in Tg mice carrying a double mutation of APP and PS1 reduced Aβ accumulation in several brain regions (Campbell et al., 2015). Intriguing findings were reported by Kvetnansky et al. (2016), who used CRF knockout mice showing that CRF potentiated tau phosphorylation during acute stress, but inhibited phosphorylation in response to repeated stress. Although the precise mechanism(s) by which CRF may exacerbate AD neuropathology remains to be determined, studies in neuronal cultures have demonstrated that CRF-induced tau phosphorylation hampers neuronal energetics and interferes with axonal transport of mitochondria (Le et al., 2016).

Tau mislocation has recently been proposed as a relevant pathophysiological mechanism in AD (Hoover et al., 2010; Tai et al., 2012; Zempel et al., 2013; Le et al., 2016). A large body of evidence suggests that hyperphosphorylated tau causes a derangement of synaptic function with a resulting impairment of excitatory synaptic transmission (Ittner et al., 2010; Crimins et al., 2013; Xie et al., 2017), leading to deficit in learning and memory (Kimura et al., 2007). In mice overexpressing APP (APP23 mice) crossed with tau transgenic mice, a redistribution of hyperphosphorylated tau from axons to dendrites increased the localization of Fyn in the postsynaptic density. Fyn, in turn, phosphorylates the GluN2B subunit of NMDA receptors at Y1472, leading to excitotoxic downstream signaling (Ittner et al., 2010). A direct effect of glucocorticoids on tau mislocation has been studied by Pinheiro and colleagues (Pinheiro et al., 2016). In male Wistar rats, prolonged (14 days) dexamethasone exposure led to cytosolic and dendritic tau accumulation in the hippocampus, but, interestingly, Fyn levels were not altered. Additional evidence of a relationship between stress-induced glucocorticoid hypersecretion and synaptic tau missorting was provided by Lopes et al. (2016), who used wild-type and tau knockout mice. In wild-type mice, exposure to CUS for 6 months caused behavioral disturbances as well as synaptic tau missorting and enhanced levels of Fyn in hippocampal postsynaptic density fractions. None of these effects were observed in mice lacking tau. Interestingly, as opposed to wild-type mice, tau knockout mice did not show changes in plasma corticosterone levels in response to CUS as well as following an acute restraint stress application. Collectively, these findings suggest that the phosphorylation status of the tau protein exerts an important role in the relationship between sustained stress and AD synaptic pathology. If chronic glucocorticoid elevation represents a causative factor contributing or exacerbating the development of AD (see also Lopes et al., 2016; Fitzpatrick et al., 2017), this would offer a pharmacotherapeutic target for AD and other tauopathies. Several studies have shown that negative experiences during childhood not only increase the probability to develop anxiety, depression, and substance use disorder but also increase the vulnerability to several clinically relevant diseases (Dich et al., 2015; Berg et al., 2017). Experimental findings support the hypothesis that early life experiences can affect adrenocortical stress response in adult life, which in turn may cause cognitive dysfunction (Chen and Baram, 2016). For the relationship between early life events and AD, see the excellent review by Lesuis et al. (2018). Here, we focus on the principal findings related to perinatal stress and AD. Exposure of pregnant APPswe/PS1dE9 mice to restraint stress during the first week of gestation caused gender-dependent behavioral and histopathological changes in the offspring. Adult male offspring showed impairment in spatial memory, while females exhibited a better performance in a spatial memory task and, interestingly, a reduced plaque load in the hippocampus (Sierksma et al., 2013). Accordingly, the effects of early life stress on the developmental trajectory of the CNS have been often reported to be gender-dependent (Naninck et al., 2015; Loi et al., 2017). In male APPswe/PS1dE9 mice, early postnatal stress (from postnatal day 2 to 9), in the form of reduced availability of bedding and nesting material, increased plaque load and impairs synaptic plasticity in the adult life (Lesuis et al., 2019). Riluzole, a drug that reduces glutamate release, prevented the effects of early life stress when added to the drinking water from weaning onwards. The effects of early life stress were also evaluated in wild-type rodents. In Wistar rats, daily maternal separation during the first 3 weeks of life induced in the adult male offspring cognitive deficits as well as increases in both Aβ40 and Aβ42 hippocampal levels in the adult male offspring. These effects were paralleled by an increased expression of BACE1 and hyperphosphorylated tau (Martisova et al., 2013). In contrast, exposure to an enriched and ‘positive’ environment during early postnatal life exerts protective effects against AD-related neuropathology and cognitive functions. In these studies, neonatal handling has been the most used experimental paradigm. Neonatal handling increases maternal care causing permanent neurochemical and behavioral alterations in the adult progeny (Meaney, 2001). Lesuis et al. (2017) have studied the effects of neonatal handling from postnatal days 2 to 9 in APPswe/PS1dE9 mice. In adulthood (11 months) mice subjected to neonatal handling showed a reduced amyloid load in the hippocampus paralleled by increased performance in learning paradigms (e.g., t-maze and contextual fear memory). In APP-V717I x Tau-T301P (biAT) bigenic mice, neonatal handling was shown to reduce hippocampal Aß accumulation and to prolong lifespan (Lesuis et al., 2016). Finally, 3xTg-AD mice daily handled from birth to weaning (postnatal day 21) showed reduced deficits in spatial learning and exploratory behavior (Cañete et al., 2015).

## Conclusions

**Figure 1:**
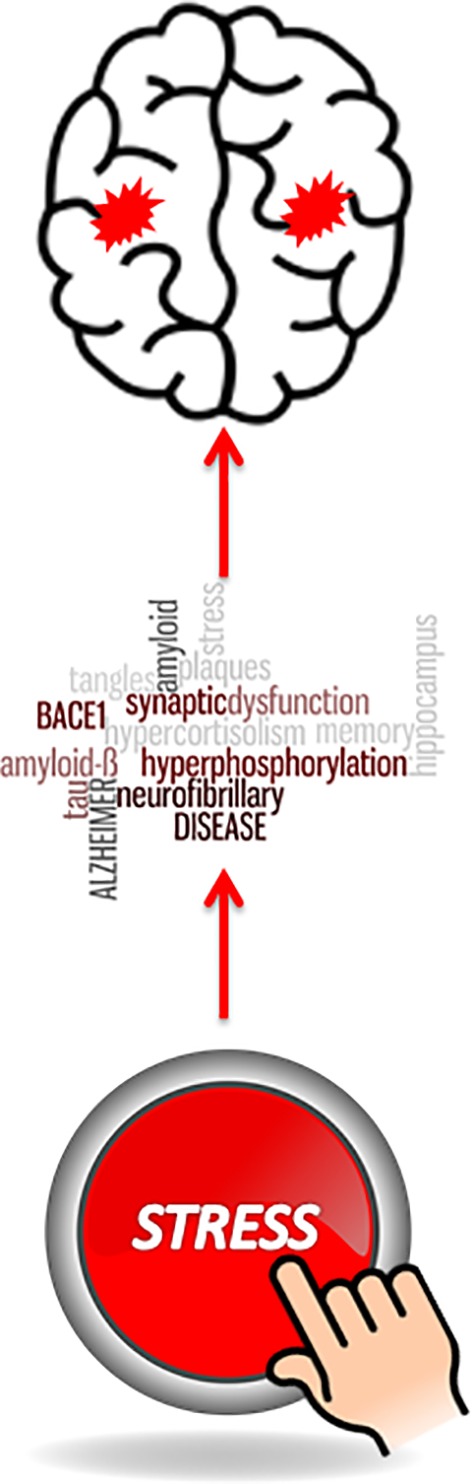
In vulnerable individuals, stress increases the risk to develop Alzheimer’s disease.

In the last years, our knowledge on the pathogenetic mechanisms of AD has dramatically improved. Several preclinical studies have demonstrated that stress is a potential risk factor for AD (**Figure 1**). However, the marked individual difference in perceiving and coping with stress makes any generalization difficult at the moment. Nevertheless, we suggest that behavioral, psychological, or pharmacological strategies aimed at increasing resilience to stress might delay the onset or slow the progression of AD.

## Author Contributions

SS, AG, and AC prepared the draft of the manuscript. FN reviewed the manuscript.

## Conflict of Interest Statement

The authors declare that the research was conducted in the absence of any commercial or financial relationships that could be construed as a potential conflict of interest.
